# Pneumococcal neuraminidase activates TGF-β signalling

**DOI:** 10.1099/mic.0.000511

**Published:** 2017-07-28

**Authors:** Nina Gratz, Lip Nam Loh, Beth Mann, Geli Gao, Robert Carter, Jason Rosch, Elaine I. Tuomanen

**Affiliations:** ^1^​ Departments of Infectious Diseases, St. Jude Children’s Research Hospital, Memphis, TN 38105, USA; ^2^​ Computational Biology, St. Jude Children’s Research Hospital, Memphis, TN 38105, USA

**Keywords:** pneumococcus, neuraminidase, TGFβ, meningitis

## Abstract

Neuraminidase A (NanA) is an important virulence factor that is anchored to the pneumococcal cell wall and cleaves sialic acid on host substrates. We noted that a secreted allele of NanA was over-represented in invasive pneumococcal isolates and promoted the development of meningitis when swapped into the genome of non-meningitis isolates replacing cell wall-anchored NanA. Both forms of recombinant NanA directly activated transforming growth factor (TGF)-β, increased SMAD signalling and promoted loss of endothelial tight junction ZO-1. However, in assays using whole bacteria, only the cell-bound NanA decreased expression of ZO-1 and showed NanA dependence of bacterial invasion of endothelial cells. We conclude that NanA secretion versus retention on the cell surface does not influence neurotropism of clinical isolates. However, we describe a new NanA-TGF-β signalling axis that leads to decreased blood-brain barrier integrity and enhances bacterial invasion.

## Introduction

*Streptococcus pneumoniae* is one of the leading causes of bacterial meningitis in young children and adults with a fatality rate up to 37 % and development of long-term sequelae in half of the survivors [1]. Meningitis occurs when the pneumococcus breaches the blood-brain barrier (BBB), a highly selective vascular network of endothelial cells that protects the central nervous system (CNS). Bacterial multiplication within the cerebrospinal fluid (CSF) leads to inflammation and damage of brain tissue. There is significant variability between clinical pneumococcal strains in the ability to reach the CNS [2] and the factors underlying this difference are poorly understood.

Neuraminidase activity has been implicated in many interactions of pneumococci with host epithelial and endothelial cells including the BBB. *S. pneumoniae* expresses three distinct neuraminidases, NanA, NanB and NanC, but only NanA is found in all pneumococcal strains [3]. NanA cleaves α2,3 and α2,6-linked sialic acids from eukaryotic and prokaryotic glycoconjugates and NanA activity reveals receptors for colonization of the upper airway [4], promotes biofilm formation [5–7], provides a carbon source for bacterial growth [8], increases virulence through sensing of sialic acid via CiaH/R [9] and enhances competition with other bacteria [10]. In a mouse model of pneumococcal infection, NanA promotes bacterial spread from the nasopharynx to the lung [2, 11]. Further, neuraminidase takes part in the evasion of opsonization and subsequent neutrophil-mediated killing [12] and secretion of proinflammatory cytokines by human leukocytes [13]. In the case of the BBB, NanA is necessary and sufficient to promote pneumococcal adherence to and invasion of BBB cells in culture [14]. Independent of sialidase activity, the laminin G-like lectin-binding domain of NanA participates in bacterial penetration [14] and induces secretion of chemokines (such as IL-8, CXCL-1 and CXCL-2) from endothelial cells [15]. For this study, we focused on the ability of neuraminidases, such as those from influenza virus and *Clostridium perfringens*, to activate latent transforming growth factor-β (LTGF-β) [16]. TGF-β is a multipotent cytokine that virtually all cells can produce and respond to. It is synthesized as pro-TGF-β that is cleaved to produce the biologically inactive complex known as LTGF-β formed by the non-covalent association of latency-associated peptide (LAP) with the dimeric mature TGF-β [17, 18]. Neuraminidases activate LTGF-β by enzymatic removal of sialic acids from LAP. In turn, activation of TGF-β signalling correlates with the invasion of respiratory epithelial cells by pneumococci [19] and BBB cells by *Escherichia coli* [20] by increasing tight junction permeability. We sought to establish if NanA triggers a TGF-β signalling cascade during pneumococcal interactions with the BBB. Further, we focused on the role of the differential activity of the previously unstudied, truncated form of NanA that is secreted from the neurotropic TIGR4 strain in comparison to the cell wall-anchored form found in the non-neurotropic D39 strain as a possible explanation of strain-dependent neurotropism.

## Results

### NanA is found in two forms: secreted and cell-bound

**Fig. 1.**
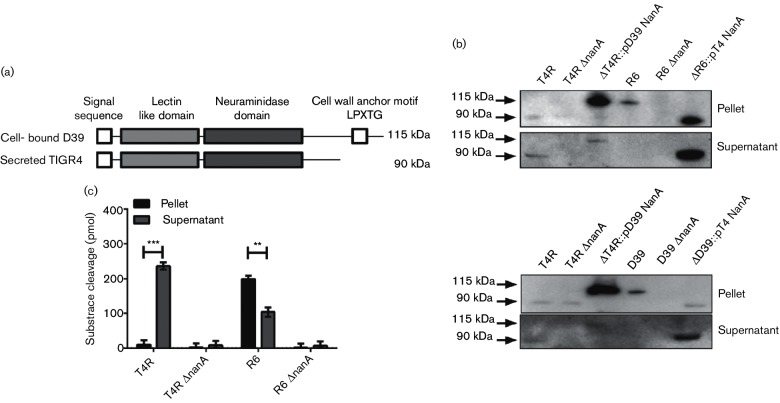
NanA exists in cell-bound and secreted forms. (a) Schematic representation of NanA from *S. pneumoniae* strains D39 and TIGR4. (b) Western blot detecting NanA in the culture pellet and supernatant of *S. pneumoniae* strains. Top panel: T4R: unencapsulated TIGR4 lacking LPXTG, R6: unencapsulated D39 with LPXTG present and their isogenic Δ*nanA* mutants. ΔT4R :: pD39 NanA: NanA with LPXTG motif introduced into the TIGR4 Δ*nanA* background; ΔR6 :: pT4 NanA: secreted NanA was introduced into the D39 Δ*nanA* background. Lower panel: the same as the upper panel except strains were encapsulated. Representative blot of three independent experiments. (c) Neuraminidase activity in the culture pellet and supernatant of T4R, R6 and their isogenic Δ*nanA-*mutants. Enzymatic activity was measured by cleavage of a fluorogenic substrate (MUNANA). Mean±sem of three independent experiments. **P=0.007; ***P=0.0001.

Most studies of the role of NanA during infection have been conducted with the D39 strain that shows poor neurotropism [2]. We noted that NanA from the TIGR4 meningitis strain differed from its homologue in D39 by the absence of a cell wall anchoring LPXTG motif due to a frame-shift mutation introducing a premature stop codon at AA801 (Fig. 1a). Rather than being a pseudogene, it has been hypothesized that both alleles of *nanA* could be transcribed but result in different localization of the protein [8]. To compare these variants a panel of mutants was created swapping the various *nanA* genes into the two different backgrounds: TIGR4 (neurotropic), D39 (non-neurotropic), their isogenic *nanA-*null mutants (TIGR4 Δ*nanA* and D39 Δ*nanA*) or each knockout strain expressing NanA from the other strain (ΔD39 :: pT4 NanA or ΔT4 :: pD39 NanA). Truncated NanA was found to be smaller in molecular size and secreted into the supernatant when expressed in either TIGR4 or D39 backgrounds (Fig. 1b). Conversely, the surface-bound version of NanA was detected in the pellet fraction in both the unencapsulated D39 (R6) and TIGR4 (T4R) backgrounds (Fig. 1b); this was also true in the encapsulated TIGR4 and D39 variants (Fig. 1b lower panel). Both alleles encode proteins that were enzymatically active as measured by cleavage of a fluorogenic substrate (Fig. 1c). Total neuraminidase activity did not differ between strains based on location of the protein (Fig. 1c).

To assess the frequency of the truncated variant of NanA in the general pneumococcal population, the presence/absence of the sequence encoding the LPXTG motif in *nanA* was ascertained in the published full genome sequencing data from 615 predominantly nasopharyngeal clinical isolates [21, 22] and 38 clinical strains from blood and CSF from the Centers for Disease Control. Within the nasopharyngeal isolates, only 1.1 % (7 of 615) lacked the LPXTG motif in the predicted NanA protein. In contrast, sequencing revealed that 31 % (12 of 38) of the invasive strains isolated from blood (7 out of 19) and CSF (5 out of 19) lacked the LPXTG motif. These findings suggested that, while cell wall-anchored NanA appears to predominate in the general pneumococcal population, the secreted version of NanA might characterize an invasive subset of isolates.

### NanA activates LTGF-β and induces TGF-β cell signalling

**Fig. 2.**
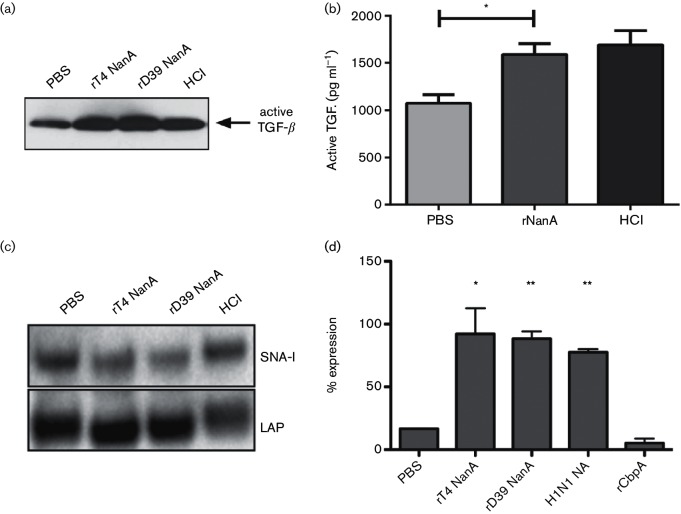
Activation of TGF-β by recombinant NanA. (a) Western blot for active TGF-β after LTGF-β was incubated with PBS, recombinant TIGR4 NanA or D39 NanA (both 1 µg) or hydrochloric acid (HCl, positive control). Representative blot from three independent experiments. (b) ELISA showing the amount of active TGF-β after stimulation of LTGF-β with PBS, 10 ng recombinant TIGR4 NanA (truncated form) or HCl (positive control). Data presented as mean±sem of three experiments. *P=0.015. (c) Quantitation of LAP after treatment of LTGF-β. 100 % was set using HCl as a maximum control for neuraminidase-independent activation. PBS is the vehicle control; influenza H1N1 neuraminidase is the positive control. Recombinant TIGR4 and D39 NanA neuraminidases are compared to pneumococcal CbpA protein (negative control). *=0.046, **=<0.006. (d) Recombinant LTGF-β was incubated with PBS, recombinant TIGR4 and D39 NanA or HCl (non-enzymatic activation with no change in lectin-binding), transferred onto a PVDF-membrane and the presence of sialic acids on the LAP was detected using HRP-coupled SNA-I lectin. The membrane was reprobed with an antibody against LAP to document loading. Representative of three independent experiments.

To investigate if *S. pneumoniae* NanA activates TGF-β, recombinant LTGF-β was incubated with recombinant full-length (D39) or truncated NanA (TIGR4) and activation of TGF-β was analysed by Western blot. Incubation of LTGF-β with both forms of recombinant NanA resulted in increased amounts of active TGF-β (Fig. 2a). Similar results were obtained when active TGF-β was measured using ELISA (Fig. 2b). NanA has been described to cleave α2,3- and α2,6-linked sialic acids from proteins [23]. We determined if conversion of LTGF-β to its biologically active form occurred by removal of sialic acids from the LAP. Incubation of LTGF-β with recombinant TIGR4 or D39 NanA resulted in a slight shift in mobility of the LAP protein to a smaller size (Fig. 2c). Using *Sambucus nigra* agglutinin (SNA-I) which recognizes α−2,6-linked sialic acid, the NanA-treated samples were shown to have reduced SNA-binding (Fig. 2d) consistent with removal of sialic acid from the LAP. Hydrochloric acid (HCl) was used as an example for non-enzymatic TGF-β activation and did not show any changes in lectin-binding. Based on these results, we concluded that the enzymatic activity of pneumococcal NanA directly converts LTGF-β into its active form by removing sialic acid from the LAP.

**Fig. 3.**
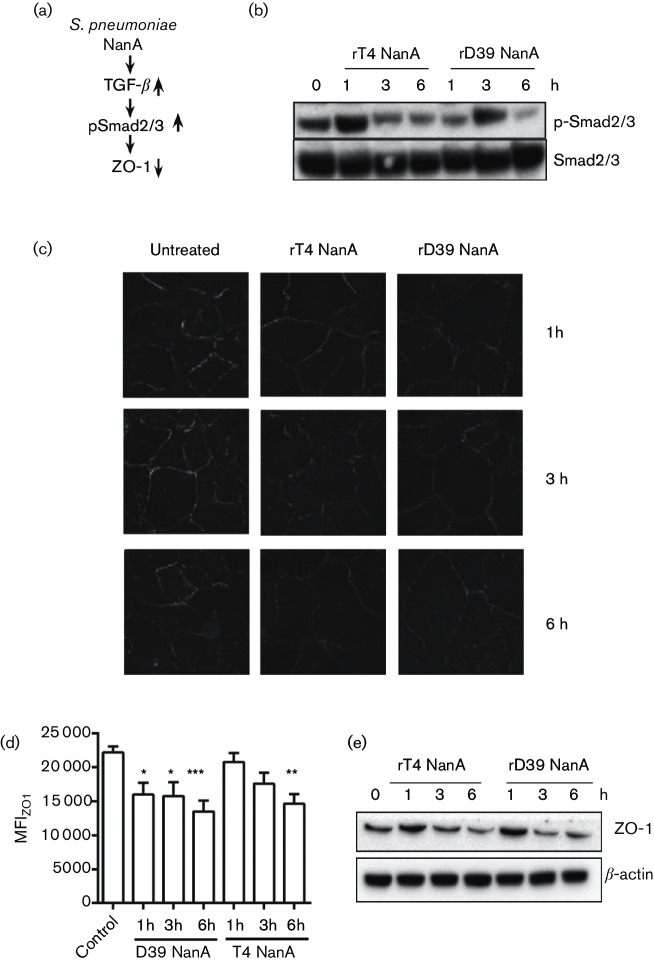
Activity of recombinant NanA on host barrier cells. (a) Scheme of the proposed signalling pathway linking NanA-mediated activation of TGF-β to decreased expression of tight junction proteins. (b) Western blot showing phosphorylation of SMAD2 and SMAD3 proteins after stimulation with recombinant TIGR4 and D39 NanA for 1, 3, 6 h in hBMECs. Equal loading was confirmed using total SMAD2/3. Representative of three independent experiments. (c) Confocal imaging showing expression of ZO-1 in endothelial hBMECs after treatment with recombinant full-length (D39) or truncated (TIGR4) NanA for 1, 3 and 6 h. Representative images from three independent experiments. Magnification: ×63. (d) Quantitation of panel A. *P<0.05, **P<0.01, ***P<0.001. (e) Western blot showing tight junction protein ZO-1 in hBMECs after stimulation with recombinant TIGR4 or D39 NanA proteins (1 µg ml^−1^) for indicated time points. β-actin served as a loading control. Representative of three independent experiments.

TGF-β signalling proceeds through phosphorylation of SMAD proteins, upregulation of the transcriptional repressor Snail1 and downregulation of tight junction proteins (Fig. 3a) [17]. Snail1 has been shown to be upregulated in the brain endothelium in response to several bacteria, including the pneumococcus, although the trigger for these responses is unknown [24]. Stimulation of hBMECs with full-length (D39) or truncated recombinant NanA (TIGR4) led to phosphorylation of the cytoplasmic SMAD2 and SMAD3 proteins (Fig. 3b). Activation of the TGF-β signalling cascade is known to progress to a decrease in tight junction proteins such as ZO-1 (18,19). Immunofluorescence microscopy of BBB endothelial cells showed that recombinant NanA, either truncated or full-length, significantly reduced expression of ZO-1 (Fig. 3c,d). Reduction of ZO-1 protein levels was also observed by Western blot in hBMECs (Fig. 3e). Thus, both forms of pneumococcal NanA can act as a trigger activating the TGF-β cascade.

### Role of NanA activation of TGF-β in bacterial interactions with the BBB *in vitro*

**Fig. 4.**
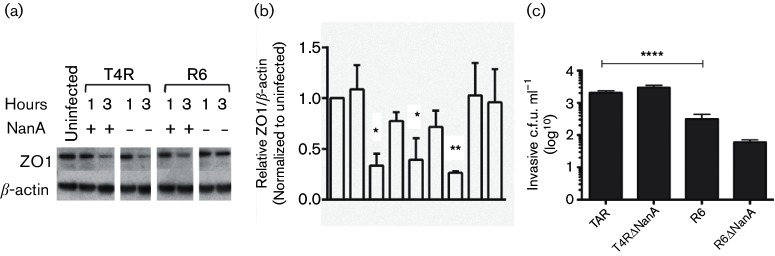
Effect of bacteria ± NanA on host barrier cells. (a) Top panel: ZO-1 expression in hBMECs after infection with T4R, T4R Δ*nanA*, R6 and R6 Δ*nanA* strains for 1 and 3 h. Data presented as mean±sem of three independent experiments. (b) quantitation of panel (a). Expression levels were normalized to uninfected control. *P<0.05, **P<0.01. (c) hBMECs were infected with the indicated pneumococcal strains and bacterial invasion was assessed by intracellular c.f.u. at 2 h. Mean±sem of ≥three independent experiments. ****P=0.0001.

Having established that recombinant NanA ± LPXTG activated TGF-β with subsequent loss of ZO-1 in endothelial monolayers *in vitro*, we sought a differential effect of NanA secretion on ZO-1 expression on bacterial invasion of hBMEC cells *in vitro*. Downregulation of ZO-1 was detected upon exposure to bacteria harbouring either the secreted or cell-bound NanA but the response was eliminated only when cell-bound NanA was deleted (Fig. 4a). Therefore, we concluded that although both forms of recombinant NanA can activate TGF-β signalling, only the cell wall-bound form appears to have an essential role in the response of tight junctions of host cells to intact pneumococci. This correlated with bacterial invasion (Fig. 4b) where the loss of cell-bound NanA in R6 resulted in a decrease of one log in bacterial invasion while deletion of secreted NanA in T4R did not affect invasion. Thus, the greater neurotropism of TIGR4 was not explained by NanA-TGF-β signalling effects on either loss of tight junctions or bacterial invasion of BBB cells.

### Role of NanA in invasion of the BBB *in vivo*

**Fig. 5.**
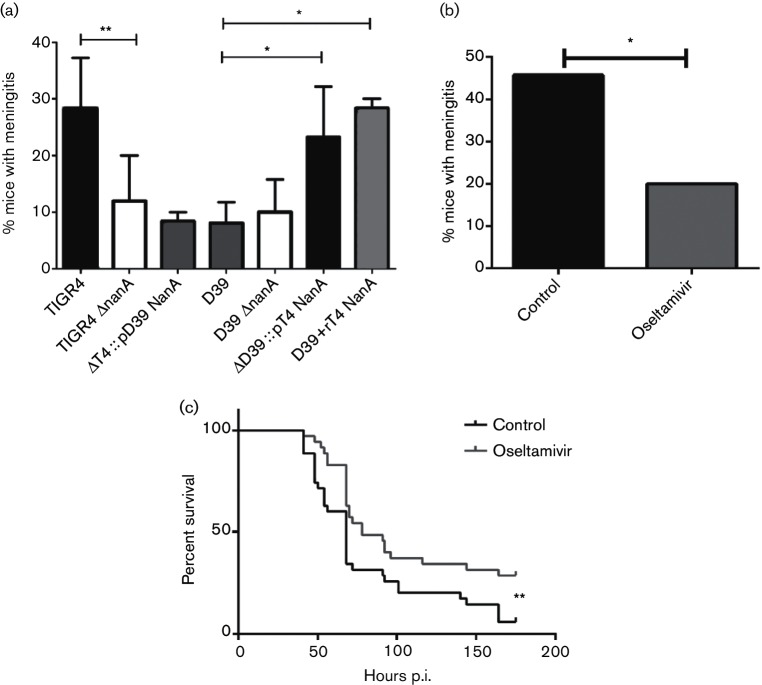
Impact of secreted NanA on experimental meningitis. (a) Prevalence of meningitis within 48 h after intravenous infection of mice with strains bearing secreted NanA (black), cell-bound NanA (grey) or deleted NanA (white). The far right grey stippled bar represents D39 supplemented with recombinant-secreted NanA given intravenously at 6 and 24 h (*n*=25). TIGR4 (*n*=52), TIGR4 Δ*nanA* (*n*=62), ΔT4 :: D39 NanA (*n*=25), D39 (*n*=45), D39 Δ*nanA* (*n*=30) and ΔD39::T4 NanA (*n*=35). Values are % of mice with positive CSF culture (values are mean±sd from at least three independent experiments). *P<0.01. (b) Incidence of meningitis in control- and oseltamivir-treated mice (*n*=35 per group). Data are presented as % mice that developed meningitis (*n*=combined from three independent experiments). *P=0.041. (c) Survival of control- and oseltamivir-treated mice after intratracheal infection with TIGR4. Treatment was given at days −1, and 1–4 post infection (*n*=35 mice per group, *n*=combined from three independent experiments). **P=0.008.

An elevated level of neuraminidase in the CSF correlates with a higher morbidity and mortality in patients suffering from meningitis [25]. To determine the importance of different forms of NanA in meningitis, Balb/c mice were challenged intravenously with TIGR4 (neurotropic), D39 (non-neurotropic), their isogenic *nanA-*null mutants or each knockout strain expressing NanA from the other strain. All mice had comparably high titre bacteremia (>10^6^ c.f.u. ml^−1^ blood) after 24 h of infection, a prerequisite for developing meningitis. The presence of bacteria in the CSF was measured at 24 and 48 h by culture. Strains carrying secreted NanA on either the TIGR4 or D39 backbone (TIGR4 and ΔD39 :: pT4 NanA) were significantly more efficient in causing meningitis than any strain with D39 (D39 and ΔT4 :: p D39 NanA) or deleted NanA (TIGR4 ΔnanA and D39 ΔnanA) (Fig. 5a). Taken together these results suggested that the secreted NanA promoted meningitis more strongly than the cell-bound form.

To further investigate the association of secreted NanA with neurotropism, animals were challenged intraperitoneally with D39 and then treated with intravenous recombinant-secreted NanA to ascertain if this would increase the incidence of meningitis (Fig. 5a). Significantly more animals developed meningitis in the rNanA-treated group indicating that the enhancement effect could be disassociated from the bacteria, potentially suggesting an effect on host cells.

The neuraminidase inhibitor oseltamivir is active against the pneumococcal neuraminidase [26]. The impact of inhibition of neuraminidase activity on meningitis was tested by pretreating mice with oseltamivir beginning 1 day prior to TIGR4 challenge. Oseltamivir treatment was continued for 4 days and mice were monitored for survival and neurological signs of meningitis for 1 week. Oseltamivir-treated mice showed significantly less meningitis compared to control mice (Fig. 5b) and demonstrated a modest but significant survival benefit despite ongoing bacteremia (Fig. 5c). These results supported the impact of NanA sialidase activity on the pathogenesis of meningitis *in vivo* and suggested that the secreted form contributes to greater pathogenicity.

## Discussion

In this study, we investigated the hypothesis that pneumococcal NanA plays a role in neurotropism during the development of meningitis. Pneumococcal NanA exists in two forms with differing localization, either attached to the cell surface in the blood isolate D39 or secreted into the culture supernatant in the meningitis isolate TIGR4. By sequence analysis, the secreted form was more rare in the general pneumococcal population while more frequent in clinical meningitis isolates. By swapping the localization of NanA in the two backgrounds, animal experiments showed the secreted form was more efficient in causing meningitis. To develop evidence for a potential mechanism linking NanA and bacterial penetration into the CSF, we linked two observations from the literature related to the respiratory tract and applied them to the BBB: influenza neuraminidase activates TGF-β [16] and activated TGF-β leads to the disruption of respiratory epithelial barrier integrity [19]. We demonstrated that recombinant pneumococcal NanA activated TGF-β signalling and consequently resulted in loss of tight junction protein ZO-1 in BBB endothelial monolayers *in vitro*.

The *in vitro* results using recombinant NanA suggested that NanA might enhance bacterial penetration of the BBB by activating the TGF-β-ZO-1 axis. However, this was contradicted by the observation that bacterial invasion and expression of ZO-1 were not dependent on NanA unless the enzyme was localized to the bacterial surface. TIGR4 showed a higher baseline level of invasion than D39 but loss of secreted NanA did not decrease invasion. Cell-bound NanA played a role in D39 penetration of the BBB in a TGF-β-ZO-1-dependent manner but, contrary to expectation, this resulted in less meningitis *in vivo* compared to NanA-independent TIGR4. Thus, there appeared to be different NanA interactions with the BBB between D39 and TIGR4. Loss of ZO-1 would be expected to open a paracellular route of bacterial translocation. This has been described for pneumococci at the respiratory epithelium where it is initiated by TLR signalling [19]. A similar TLR2-dependent paracellular pathway has been described at the BBB for Group B streptococci [25]. However, pneumococci have not been shown to use a paracellular route across the BBB and involvement of NanA in any such paracellular pathway for any bacteria has not been described. Rather, transcellular pathways, for instance receptor-mediated uptake involving laminin receptor and PAFr or receptor-independent macropinocytosis, characterize BBB translocation of pneumococci [27–29]. It appears that NanA in either form does not influence these transcellular events even if it contributes to low-level translocation of pneumococci between cells in a cell-bound form.

It should be noted that NanA has two modes of interaction with human cells [14]: sialidase activity and via lectin activity. The lectin activity predominates during bacterial adherence and invasion by creating a physical link between bacteria and host cells. This is consistent with the findings here where cell-bound NanA showed a greater impact on BBB interactions when displayed on whole bacteria. The mechanism by which secreted sialidase activity enhances neurotropism *in vivo* appears to be directed to effects on host cells.

In conclusion, we have presented evidence that formulates a signalling pathway whereby pneumococcal neuraminidase induces TGF-β-mediated downregulation of tight junction protein expression and increased invasion of BBB endothelial cells. This effect appears to require NanA attachment to the bacterial surface. However, this pathway does not explain the greater neurotropism of strains with the secreted form of the neuraminidase or the ability of secreted NanA to enhance meningitis when supplied *in trans*. It remains to be determined which targets of sialidase activity during pathogenesis not involving attachment/invasion and barrier integrity contribute to strain-specific neurotropism.

## Experimental procedures

### *nanA* alleles in clinical strains

A total of 615 previously published *S. pneumoniae* sequences were re-examined for *nanA* [18, 19]. To identify *nanA* genes in the assemblies, each predicted coding sequence that was annotated as a sialidase was extracted and translated from each assembly. Sialidases from each sample were aligned to *S. pneumoniae* D39 and *S. pneumoniae* TIGR4 *nanA* and *nanB* sequences to identify the *nanA* orthologue in each sample. The *nanA* orthologues across all samples were combined and aligned using MAFFT. The resulting multiple sequence alignment was used to identify whether each sample contained a D39-like or TIGR4-like *nanA* orthologue.

Forty-three clinical *S. pneumoniae* strains from blood and CSF from the Centers for Disease Control were grown to the mid-log phase and genomic DNA was extracted. The *nanA* gene was amplified using PCR with primers: NanA_1: TCCCACATAAGTTCCTAAACGG and NanA_2: GTT CGATAAGGATTGAGCAGGAAG. PCR products were purified with a PCR purification kit (Qiagen) and used for further sequencing with the following primers: NGS_1: CGTATCAGCCTGAATGTCATC, NGS2: GGCACCTGCTAGATTTACAGG, NGS3: CTTCTCCCAAGTCACTCCTC, NGS4: CAACAACGCGATAGTCTGTC, NGS5: TGTTA AAGCCGCTCCTTCAG, NGS6: CTCTCTGACCATTTG AAGCATTGG, NGS7: GATTGGAGAAAGGAGAGGGG, NGS8: GCTTCAAATGGTCAGAGAGTTG. Predicted peptides were analysed for the presence of a LPXTG motif.

### Bacterial strains and growth conditions

*S. pneumoniae* strains used were the serotype 4 meningitis isolate TIGR4 and its unencapsulated derivative T4R and the serotype 2 bacteremia isolate D39 and its unencapsulated derivative R6. Bacteria were grown on tryptic soy agar (EMD Chemicals, NJ) supplemented with 3 % sheep blood or in a defined semisynthetic casein liquid medium supplemented with 0.5 % yeast extract (C+Y) [29]. Erythromycin (1 µg ml^−1^) and kanamycin (400 µg ml^−1^) were added when appropriate. Cultures were inoculated from frozen stock and incubated at 37 °C in 5 % CO_2_.

### Bacterial constructs

Deletion disruption mutations of SP_1693 (*nanA* in TIGR4) and SPD_1504 (*nanA* in D39) were created by using the splicing by overhang extension method of PCR (SOE-PCR) [30]. Approximately 1 kb upstream and downstream fragments of the target gene were amplified and spliced to an erythromycin resistance gene (*ermB*). SOE-PCR products were subsequently transformed into the pneumococcus (TIGR4, D39, T4R and R6), and all deletion disruption knockouts were verified by PCR to confirm insertion of the SOE-PCR product and deletion of the target gene [31]. The TIGR4 and D39 *nanA-*mutants were complemented with the indicated alternative *nanA* by transforming each mutant with the construct pD39 NanA or pT4 NanA. Briefly, the *nanA* coding region including a promoter sequence upstream was amplified from T4 and D39 genomic DNA using primers T4NanAPst (GCGCGCCTGCAGTTTACTAACGCTTATA AAAAGTG) and T4NanABam (GCGCGCGGATCCCTAGTTCGCTTCGGTAGGAG) for T4, and D39NanA Pst (GCGCGCCTGCAGTTTACTAACACTTATAAAAAGTG) and D39NanABam (GCGCGCGGATCCTTATTGTTCTCTCTTTTTCCC) for D39. PCR products were digested with PstI/BamHI and cloned into prepared vector pABG5mini (kind gift of Michael Caparon). Clones with the correct DNA sequence were transformed into the pneumococcal mutant strain of choice and selected with Erm/Kan. Complemented mutants were confirmed by Western blot using a polyclonal antibody against NanA and tested for enzymatic activity.

### Production of recombinant proteins

*nanA* was amplified from TIGR4 and D39 genomic DNA using primers NanANde (CGCGCGCGCATATGAATCGGAGTGTTCAAG) and NanABam (CGCC GCGGGATCCCTAGTTCGCTTCGGTAGGAG), digested with NdeI and BamHI and cloned into prepared vector pET15b. Clones with the correct coding sequence were transformed into BL21 (DE3) cells. Protein expression was induced overnight at 22 °C with 0.07 mM IPTG. Recombinant protein was purified using Ni-NTA agarose (Qiagen) and dialyzed into PBS. Endotoxin contamination in purified protein preparations was removed using high capacity Endotoxin Removal Columns and the level of endotoxin was determined using a LAL Chromogenic Endotoxin Quantitation Kit.

### Infection models

Six–eight-week-old female Balb/c mice were purchased from The Jackson Laboratory. For meningitis experiments, mice (10/group) were injected intravenously with 1×10^5^ c.f.u. of the desired strain. Twenty-four and 48 h post challenge, mice were anaesthetized, blood and CSF were collected, and serial dilutions were plated to enumerate bacterial CFUs. The presence of any bacteria on CSF culture was used as a read-out for meningitis. For some experiments, 10–15 mice were treated with intravenous recombinant TIGR4 NanA (10 ug) at 6 and 24 h after intravenous D39 challenge. Control animals (*n*=10) received D39 alone.

For oseltamivir experiments, mice were orally administered with either Tamiflu (Genentech, 50 mg kg^−1^, dissolved in autoclaved tap water) or autoclaved tap water (control) 1 day before infection. Treatment (1x/day) continued for four consecutive days. Mice were infected intratracheally with 1×10^5^ of TIGR4 and were monitored three times a day for survival and development of neurological signs of meningitis such as lethargy, head tilt, walking in circles, episthotonus and opisthotonus. Mice displaying signs of meningitis were sacrificed.

Cell culture hBMECs were grown to confluent monolayers on 60 mm dishes (Corning) and infected with unencapsulated *S. pneumoniae* (1×10^7^ c.f.u. 3 ml^−1^ medium). After indicated time-points, cells were either lysed with RIPA-buffer for Western blot experiments or TRIzol LS reagent (Invitrogen) for RNA preparation and subsequent qRT-PCR. For invasion assays, cells were grown in 24-well plates at 37 °C in 5 % CO_2_ to 80% confluency and activated for 2 h with human tumor necrosis factor alpha (TNF-α, 10 ng ml^−1^, R and D Systems). Cells were incubated with bacteria (1×10^7^ c.f.u./well of unencapsulated derivatives) for 2 h, washed three times in DPBS, and subjected to 1 h of penicillin (10 µg ml^−1^) and gentamicin (200 µg ml^−1^) to kill extracellular bacteria. The cells were again washed, trypsinized, and lysed with 0.025 % Triton X-100. Colonies were incubated overnight on blood agar plates and counted to represent the intracellular bacterial number. Four wells were used for each strain, and the assays were repeated three times.

### Neuraminidase activity assays

Neuraminidase enzymatic activity was determined by the MUNANA (2-(4-methylumbelliferyl)-a-d-N-acetylneuraminic acid) assay as described elsewhere [16]. Bacteria were grown to the mid-log phase (OD_620_ 0.4) in C+Y medium, centrifuged and supernatants were collected and kept on ice. The pellet was resuspended in enzyme buffer and sonicated for 30 s with rest intervals of 1 min for four times on ice. Overall, 20 µl of the samples were loaded on an opaque 96-well microtitre plate, mixed with 30 µl buffer containing the substrate and incubated for 1 h at 37 °C with occasional shaking in the dark. Reaction was stopped with stop solution and plate was read (extinction of 365 nm and emission of 450 nm) immediately on a Fluoroskan Ascent plate reader (ThermoScientific).

### TGF-β assays

For Western blot analysis of TGF-β activation, human recombinant latent TGF-β (1 µg, R and D Systems) was incubated with PBS, HCl (final pH of 2), 1 µg TIGR4 NanA or D39 NanA, influenza H1N1 neuraminidase (1 unit) and recombinant CbpA protein (1 µg) for 1 h at 37 °C. All reactions were performed in the presence of a protease inhibitor to prevent protease-mediated activation of LTGF-β. Samples were separated using a 4–12 % Bis-Tris gel (Novex), transferred to a PVDF-membrane and probed with an antibody against active TGF-β or LAP.

For ELISA experiments, an anti-human TGF-β DuoSet ELISA kit (R and D Systems) was used.

Recombinant LTGF-β (0.25 µg) was incubated with PBS, 10 ng recombinant TIGR4 NanA protein or HCl in the presence of a protease inhibitor for 1 h at 37 °C and immediately loaded on a ELISA plate. ELISA was performed according to the manufacturer’s instructions.

For the detection of sialic acids on the LAP, LTGF-β (1 µg) was incubated with PBS, recombinant TIGR4 or D39 NanA (both 2 µg) and HCl for 1 h at 37° C in the presence of a protease inhibitor. Samples were separated on 4–12 % Bis-Tris gel under reducing conditions, transferred to a PVDF-membrane, blocked for 1 h with RIPA buffer and probed with HRP-conjugated *Sambucus nigra* SNA-I (0.2 µg ml^−1^ in RIPA buffer, EY Laboratories) for 2 h at room temperature. After several washes in RIPA buffer, lectin-binding was detected using a chemiluminescent substrate.

### Western blot

Bacterial cultures were grown to the mid-log phase and pelleted by centrifugation and subjected to lysis in 0.1 % Triton X-100. For *in vitro* experiments, infected cells were washed once with ice-cold PBS, lysed with RIPA-buffer containing proteinase-) and phosphatase inhibitors. Lysates were centrifuged at 13 000 rpm at 4 °C for 15 min, and the supernatant was collected. Lysates were run on 4–12 % NuPage Bis-Tris gels (Invitrogen), transferred on a PVDF-membrane and probed with anti-phospho-SMAD2/3 (Santa Cruz), anti-SMAD2/3 (Santa Cruz), anti-ZO-1 (BD Transduction Laboratories) and β-actin (Sigma). Polyclonal rabbit antiserum against full-length D39 NanA was developed at Rockland Immunochemicals. Blots were imaged using a ChemiDoc MP imaging system.

Microscopy hBMECs were plated at 5×10^4^ cells per well on fibronectin-coated glass coverslips (neuVitro) 2 days prior to treatment. Cells were fixed with methanol for 10 min at −20 °C. After fixation, the cells were washed and stained with mouse anti-ZO1 (BD Transduction Laboratories) in blocking buffer (BB) (PBS containing 5 % (v/v) FBS) with 0.2 % saponin overnight at 4 °C. Cells were stained with Alexa Fluor 555 conjugated donkey anti-mouse IgG (H+L) secondary antibody (Invitrogen), washed and mounted with EverBrite mounting medium with DAPI. At least six random fields of z-stacks were acquired using a Marianas spinning disk confocal imaging system (Intelligent Imaging Innovations/3i) consisting of a CSU-22 confocal head (Yokogawa Electric Corporation, Japan), and solid-state diode lasers for various wavelengths. Images were acquired with a Zeiss Plan-Apochromat 63×1.4 NA DIC objective and a Cascade II 512 EMCCD camera using SlideBook 6 software. Mean intensity of ZO1 of at least six random fields from three independent experiments was quantified by using SlideBook 6 software.

### Statistical analysis

Statistical analysis of invasion assays, neuraminidase assays, ELISA and qRT-PCRs were performed with two-tailed unpaired parametric *t-*tests. *P-*values of 0.05 or less were considered significant: *P<0.05, **P<0.01 and ***P<0.001, ns= not significant. Survival experiments were analysed using the log rank (Mantel–Cox) test. Meningitis experiments were analysed using Fisher’s exact test (two-sided, α <0.05 was considered significant). All statistical analyses were performed using the GraphPad Prism 6 software for Windows.
